# Effects of a New Nutraceutical Formulation (Berberine, Red Yeast Rice and Chitosan) on Non-HDL Cholesterol Levels in Individuals with Dyslipidemia: Results from a Randomized, Double Blind, Placebo-Controlled Study

**DOI:** 10.3390/ijms18071498

**Published:** 2017-07-12

**Authors:** Valentina Spigoni, Raffaella Aldigeri, Monica Antonini, Maria Maddalena Micheli, Federica Fantuzzi, Andrea Fratter, Marzia Pellizzato, Eleonora Derlindati, Ivana Zavaroni, Riccardo C. Bonadonna, Alessandra Dei Cas

**Affiliations:** 1 Department of Medicine and Surgery, University of Parma, 43126 Parma, Italy; valentina.spigoni@unipr.it (V.S.); raffaella.aldigeri@unipr.it (R.A.); federica.fantuzzi@studenti.unipr.it (F.F.); eleonora.derlindati@unipr.it (E.D.); ivana.zavaroni@unipr.it (I.Z.); riccardo.bonadonna@unipr.it (R.C.B.); 2 Division of Endocrinology and Metabolic Diseases, Azienda Ospedaliero-Universitaria of Parma, 43126 Parma, Italy; monypenny1975@libero.it (M.A.); maddi.michi@alice.it (M.M.M.); 3 Nutraceutical Research and Innovation Technology, Labomar Research, Istrana, 31036 Treviso, Italy; andrea.fratter@labomar.com; 4 Nutraceutical Formulation, Labomar Research, Istrana, 31036 Treviso, Italy; marzia.pellizzato@labomar.com

**Keywords:** nutraceuticals, non-HDL cholesterol, PCSK9, randomized clinical trial

## Abstract

Increased non high-density lipoprotein (HDL)/low-density lipoprotein (LDL) cholesterol levels are independent risk factors for cardiovascular (CV) mortality with no documented threshold. A new combination of nutraceuticals (berberine 200 mg, monacolin K 3 mg, chitosan 10 mg and coenzyme Q 10 mg) with additive lipid-lowering properties has become available. The aim of the study is to test the efficacy of the nutraceutical formulation (one daily) in lowering non-HDL cholesterol vs. placebo at 12 weeks in individuals with non-HDL-cholesterol levels ≥160 mg/dL. 39 subjects (age 52 ± 11 years; 54% females; body mass index 27 ± 4 kg/m^2^) were randomized (3:1) in a double blind phase II placebo-controlled study. At baseline, 4 and 12 weeks main clinical/biohumoral parameters, pro-inflammatory cytokines, (gut)-hormones, proprotein convertase subtilisin/kexin type 9 (PCSK9) levels and endothelial progenitor cell (EPC) number were assessed. Baseline characteristics were comparable in the two groups. The intervention significantly decreased non-HDL cholesterol (−30 ± 20 mg/dL; *p =* 0.012), LDL cholesterol (−31 ± 18 mg/dL, *p =* 0.011) and apolipoprotein (Apo) B (−14 ± 12 mg/dL, *p =* 0.030) levels compared to the placebo. Pro-inflammatory, hormonal, PCSK9 and EPC levels remained stable throughout the study in both groups. The intervention was well tolerated. Three adverse events occurred: Epstein Barr virus infection, duodenitis and asymptomatic but significant increase in creatine phosphokinase (following intense physical exercise) which required hospitalization. The tested nutraceutical formulation may represent a possible therapeutic strategy in dyslipidemic individuals in primary prevention.

## 1. Introduction

Increased cholesterol levels are an established cardiovascular (CV) risk factor linearly associated with CV outcomes with no documented threshold [1,2]. In a meta-analysis including over 90,000 individuals, each 1 mmol/L (38.7 mg/dL) decrease in low-density lipoprotein (LDL)-cholesterol (LDL-C) levels with statin therapy yielded a 23% reduction in the risk of major coronary events over five years [3]. Recently, non-high-density lipoprotein cholesterol (non HDL-C) emerged as a new target for lipid lowering therapy as, compared to LDL-C, it includes all atherogenic cholesterol (triglyceride-rich lipoprotein, very low density lipoprotein, chylomicron remnants and intermediate-density lipoprotein). Accordingly, prospective studies showed that non-HDL-C is an independent risk factor for myocardial infarction [4,5], also in primary prevention [6,7,8].

The recent development of the plasma proprotein convertase subtilisin/kexin type 9 (PCSK9) inhibitors has highlighted the importance of PCSK9 as a new therapeutic target for lowering LDL-C and dyslipidemia-associated CV disease (CVD) [9]. PCSK9 is a liver protease which promotes hepatic LDL receptor degradation with consequent reduction in LDL receptor density and uptake and LDL clearance, with increased LDL-C levels [10]. Emerging evidence demonstrated that statin therapy is associated with an undesirable significant increase in plasma PCSK9 irrespective of type of statin and treatment duration, and that its combination therapy with ezetimibe may further increase PCSK9 compared to statin alone, thus blunting lipid-lowering and cardioprotective actions of statins, and, perhaps of ezetimibe [11].

Lipid-lowering properties have been claimed for some nutraceutical compounds, namely plant sterols and stanols, bile acid sequestrants, red yeast rice- and the active ingredient monacolin K- and berberine [12]. Briefly, these compounds may act with different mechanisms: (a) by inhibiting intestinal absorption of cholesterol (sterols and stanols, bile acid sequestrants) [13]; (b) by inhibiting 3-hydroxy-3-methyl-glutaryl-CoA (HMG-CoA) reductase (monacolin K) [14]; (c) by inducing an increase in LDL receptor expression and half-life and increasing insulin sensitivity via AMP-activated protein kinase (AMPK) activation (berberine) [15]. Due to their complementary and additive lipid-lowering actions, some of these compounds can be used in combination to potentiate their efficacy. No evidence, to date, is available on whether nutraceutical compounds may affect PCSK9 bioavailability [16]. Recently, a nutraceutical combination (red rice yeast extract (RRYE) titrated in monacolin (MC), berberine chloride (BC)), assembled in a new technology (named coleosoma) became commercially available (TegraDOC^®^). Briefly, it is composed of a gastro-resistant tablet containing, in the inner core, a chitosan (CH) polymer and an organic acid (phosphoserine, PS) that reduces enteric fat absorption [17]. This latter mechanism, coupled with the inhibition of HMG Co-A reductase and induced LDL-C clearance mediated by MC and BC, respectively, accounts for the lipid lowering properties of this compound.

The aim of the present study was to assess the efficacy of the above mentioned nutraceutical combination in terms of reduction in non-HDL-C and in the levels of other metabolic, inflammatory, hormonal, and vascular parameters. Specifically, as a vascular endpoint, endothelial progenitor cell (EPC) number was assessed as a biomarker of vascular damage [18].and incident CVD [19]. PCSK9 levels were also assessed. This study shows that this nutraceutical formulation was effective in the reduction of non-HDL/LDL-C levels.

## 2. Results

### 2.1. Study Subjects

**Figure 1:**
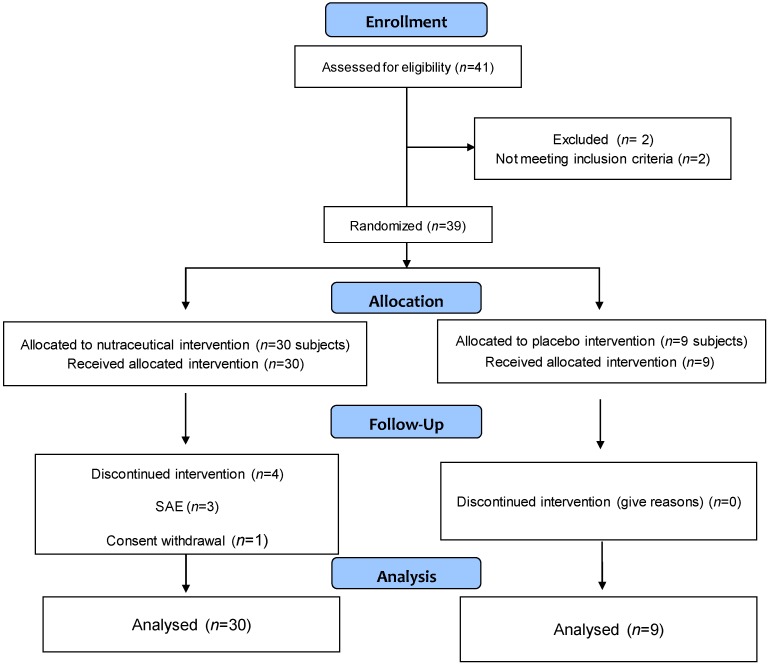
Study Consort diagram. Consort flow-chart according to the intention-to-treat analysis of the study. (SAE: severe adverse event).

41 individuals with hypercholesterolemia (non-HDL-C ≥ 160 mg/dL) were screened. Of these, 39 were randomized to receive the nutraceutical compound (*n* = 30) or placebo (*n* = 9) according to a 3:1 ratio. Four individuals in the intervention arm discontinued the intervention due to the occurrence of severe adverse events (SAE) (*n* = 3) and one subject withdrew their informed consent. Conversely, no subjects in the placebo arm discontinued the intervention in the 12 weeks of follow-up. Figure 1 shows the Consort flowchart according to intention to treat (ITT) analysis. The median duration of therapy was 12 (inter quartile range (IQR): 11–13) weeks, with a treatment adherence, assessed by pill count, of 94 ± 6%, without significant difference between study arms. Baseline characteristics of the study subjects did not differ in the two study groups as reported in Table 1. Concomitant medications and comorbidities were equally distributed in the two study arms (Table 1).

### 2.2. Effect of Treatment on Primary Endpoint

**Figure 2:**
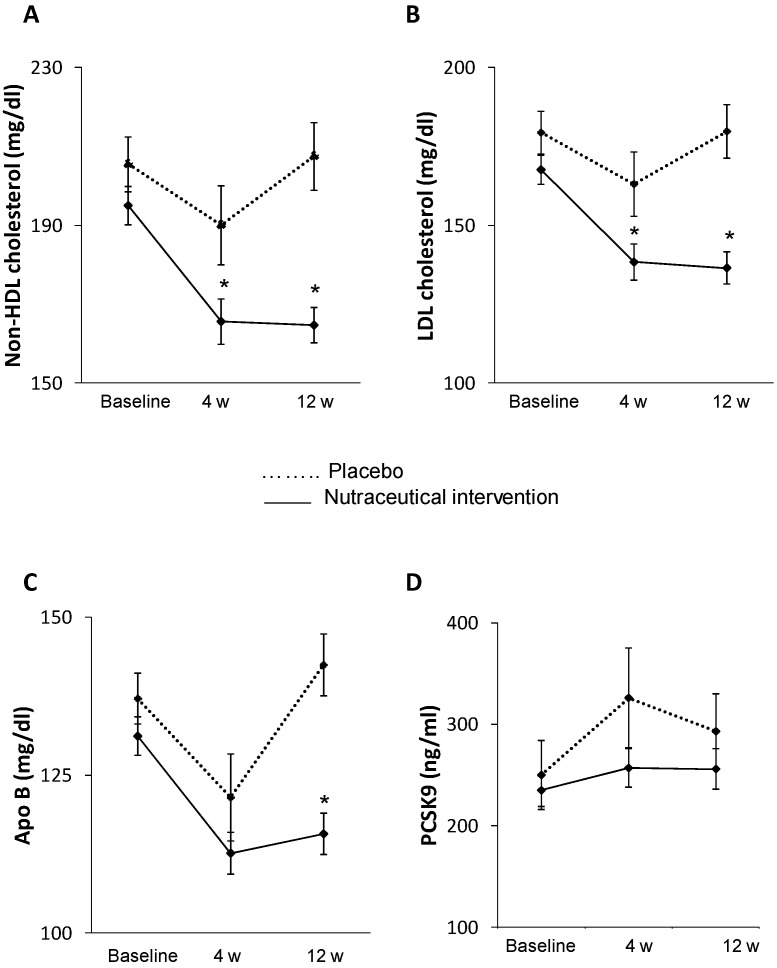
Lipid levels at baseline, 4 and 12 weeks of treatment in both study arms. Effect of treatment on non-HDL-cholesterol (**A**), LDL-cholesterol (**B**), Apo B (**C**) and PCSK9 (**D**) levels at 4 and 12 weeks. Mean values ± standard error (SE) are presented. * *p* < 0.05 vs. placebo (GLM: general linear model) (HDL: high-density lipoprotein; LDL: low-density lipoprotein, Apo: apolipoprotein, PCSK9: Proprotein convertase subtilisin/kexin type 9).

After 12 weeks, the nutraceutical compound significantly reduced non-HDL-C by 15.1 ± 1.7(SE)% compared to the placebo (−1.1 ± 3.4%). Non-HDL-C significantly decreased during follow-up (*p* < 0.0001) and in the active treatment arm with respect to placebo (β = −42.8 ± 9.4, *p* < 0.0001) as shown in Figure 2A. There was a significant correlation between baseline level of non-HDL-C and the reduction observed after 12 weeks of treatment (*r* = 0.474, *p =* 0.008).

### 2.3. Effect of Treatment on Secondary Endpoints

#### 2.3.1. Effect on Metabolic Parameters

Non-HDL-C reduction was also confirmed after four weeks of treatment (15.0 ± 2.1% vs. 7.6 ± 3.3%; general linear model (GLM) *p =* 0.047, Figure 2a). This significant decrease was mirrored by a similar significant reduction in LDL-C (−19 ± 2.0% vs. −4 ± 3.9%; GLM *p =* 0.008; Figure 2b) and Apolipoprotein (Apo) B (−12 ± 1.7% vs. −4 ± 1.2%; GLM *p =* 0.023; Figure 2c) in the nutraceutical arm compared to placebo at 12 weeks of treatment. A significant decrease in LDL-C was also observed after four weeks of treatment (−17.5 ± 2.5% vs. −3.8 ± 1.2%; GLM *p =* 0.044; Figure 2b). No changes were observed between treatment arms in HDL-C, triglycerides, fasting plasma glucose (FPG), glycated haemoglobin (HbA1C), waist circumference and body mass index (BMI) levels (Table 2). Differences in ApoB/ApoA ratio did not reach statistical significance (*p =* 0.07).

#### 2.3.2. Effect on Inflammatory Markers and Hormone Profile

Inflammatory markers and hormones were stable throughout the study in both groups (Table S1).

#### 2.3.3. Effects on PCSK9

No significant increase in PCSK9 levels were observed during the follow-up (GLM *p* = 0.092) or between treatments (GLM *p =* 0.244; Figure 2d).

#### 2.3.4. Effects on EPC Number

No changes in EPC number occurred at 12 weeks in both study arms (Table S2).

#### 2.3.5. Adverse Events and Safety Assessment

Three out of 30 (10%) subjects in the nutraceutical arm discontinued intervention. One subject experienced a duodenitis at week 10 and another presented a myocarditis (at week 8) as a result of an Epstein-Barr virus infection. In one subject, an asymptomatic but significant increase in creatine phosphokinase (CPK) (following intense physical exercise) which required hospitalization was detected at V2 (week four) and completely resolved in two days following hydration. No adverse events were reported in the placebo arm during the whole study period. Specifically, no changes in hepatic and renal function and CPK levels were observed during the study in either of the arms, as shown in Table 3.

## 3. Discussion

This double blind, randomized placebo-controlled study shows that a 12-week treatment with a newly available nutraceutical formulation—containing a combination of compounds with putative complementary lipid-lowering properties (namely chitosan, red yeast rice, and berberine)—was effective in reducing plasma non-HDL-C and LDL-C compared to the placebo, without affecting PCSK9 bioavailability in individuals with hypercholesterolemia. Considering the compelling effectiveness of reducing non-HDL/LDL-C to prevent CV events and the negative role played by an increased expression of PCSK9, we can speculate that this compound may represent a useful strategy for individuals in primary prevention.

Non-HDL-C reflects the full burden of the cholesterol transported in atherogenic lipoproteins and its assessment for CVD risk prediction and as a target of therapy has been emphasized in several guidelines [20,21]. Non-HDL-C was closely associated with plaque progression [22] and was more predictive of the severity of coronary atherosclerosis compared to LDL-C [23]; this is also true in primary prevention studies [6,7,8]. Among statin-treated patients, on-treatment levels of non-HDL-C were more strongly associated with the risk of future major CV events compared to LDL-C and ApoB in a large meta-analysis [24]. In our study, the tested nutraceutical compound was effective in reducing non-HDL-C by ~15% (as well as LDL-C by 20%) (corresponding to ~30 mg/dL) at 12 weeks of treatment. As expected, this effect was greater in those with higher cholesterol levels at baseline. Accordingly with LDL-C reduction, a similar decrease in ApoB concentrations was observed. Available evidence shows that ApoB-100 encloses all atherogenic lipoproteins and is a reliable predictor, together with the ApoB/ApoA1 ratio, of CVD and stroke risk [25,26,27]. Although in our study ApoB/ApoA1 ratio tended to be reduced, the statistical significance was not reached. The extent of non-HDL/LDL-C reduction observed in our study is comparable to that achieved with low potency statins (i.e., pravastatin, lovastatin), low doses of simvastatin (i.e., 10 mg) or ezetimibe alone [28]. The extent of cholesterol decrease is in line with that also obtained with the most studied nutraceutical combination approach (policosanol 10 mg, BC 500 mg and MC 3 mg) as shown in a 12 week randomized double-blind study [29]. Accordingly, in a recent review of the clinical evidence, the reduction in LDL-C with the use of the above mentioned formulation, ranges from 15 to 31% with an effect which was clearly proportional to starting values [30].

It is important to note that in contrast with statin therapy, the lipid lowering effect obtained with the nutraceutical compound was not associated with an increase in PCSK9 levels. This might be particularly desirable as PCSK9 is also expressed in human atherosclerotic plaque nurturing foam cell formation and atherogenesis and seems to be associated with an early marker of cardiovascular risk [31,32]. Statin-induced PCSK9 increase may partially explain the limitation to reach therapeutic targets and the need to progressively augment statin dose at the expense of increased risk of myopathies [11]. The lack of increase in PCSK9 levels might suggest the hypothesis of a more predictive dose-response and durable effect of the studied nutraceutical formulation in lowering lipid levels.

Berberine is known to also retain favourable insulin-sensitising effects through the activation of the intracellular AMPK signalling. However, the effects of berberine on glucose homeostasis are conflicting. In some studies conducted with 500 mg/day of BC [33] insulin, glucose and HOMA-IR (homeostasis model assessment-insulin resistance) values did not change. In other studies that [34,35] include individuals with insulin-resistance/metabolic syndrome, leptin-to-adiponectin ratio and the prevalence of metabolic syndrome was significantly lower in subjects treated with BC. We cannot exclude that the inclusion in our study of dysmetabolic subjects might have led to different results. Accordingly, no changes in (entero) hormone, assessed as a potential mechanism underlying a possible amelioration in insulin/glucose homeostasis,s were observed. To the best of our knowledge, no studies have investigated the entero-axis response to nutraceuticals so that future studies are warranted to definitively rule out a possible effect of the nutraceutical compounds in modulating (gut) hormone secretion. Two additional findings of our study deserve to be discussed.

Pro-inflammatory parameters remained unchanged during the study. It is known that cholesterol reduction with statins is accompanied by a reduction in low-grade inflammation [36]. Anti-inflammatory effects of nutraceutical compounds have generally been observed, although studies have yielded different results. In a recent meta-analysis, regular intake of phytosterols-enriched foods did not significantly change C-reactive protein (CRP), whilst LDL-C concentrations were significantly reduced [37]. On the contrary, the LDL-C reduction of 30% with the use of the nutraceutical combination of berberine 500 mg, silymarin 105 mg from Silybum marianum, and monacolin K 10 mg (the equivalent of a dose of lovastatin 10 mg) was paralleled by a significant reduction in CRP and interleukin (IL)-6 [38], whereas in a double-blind and crossover study no effect on inflammation was observed despite a reduction in lipid levels with a combination of red yeast rice extract, berberine, policosanol, astaxanthin, coenzyme Q10, and folic acid [34]. Again, we cannot exclude that the inclusion in the study of a higher risk population (presumably with a higher degree of low-grade inflammation) might have led to different results.

EPCs are key players in the process of endothelial repair/replacement as they substantially contribute to endothelial homeostasis and neoangiogenesis in response to different detrimental cues [13]. Fewer EPCs are a biomarker, and perhaps a pathogenetic factor, of vascular damage. In contrast to a previous report which showed that BR 1200 mg/day induced upregulation of the number and function of circulating EPCs in healthy subjects by enhancing nitric oxide production [39], no changes in circulating EPCs were observed.

The nutraceutical compound was well-tolerated as demonstrated by the lack of changes in the hepatic and renal profiles and CPK levels in the treatment compared to the placebo arm. However, three SAE occurred in the intervention arm: Epstein Barr virus infection and duodenitis, which were not related to the study compound. On the contrary, the nutraceutical compound may have amplified CPK increase following a vigorous physical exercise which was treated by hydration with complete resolution, but which required hospitalization.

The strength of our study includes the controlled-randomized double blind study design which ensured the clear demonstration of the lipid lowering efficacy of the nutraceutical treatment. Some limitations of our study should be acknowledged. This was a short-term study in a selected dyslipidemic population in primary prevention so that study results should not be generalized to other populations. In addition, the lack of a group treated solely with CH polymer does not allow the discrimination of the lipid lowering effects owed to a reduction in enteric fat absorption to those due to the inhibition of HMG Co-A reductase and induced LDL-C clearance mediated by the other components (MC, BC, Q10).

## 4. Materials and Methods

We conducted a single-centre phase II, randomized (3:1), double blind, placebo-controlled study (ClinicalTrials.gov identifier NCT03027336) to compare the efficacy of a new nutraceutical formulation once daily vs. placebo in reducing non-HDL-C levels at 12 weeks of treatment. The study was conducted according to the guidelines of Good Clinical Practice and the Declaration of Helsinki; the protocol and amendments were approved by the Parma Ethics Committee, reference number: 2015-n 42174 (21 September 2015). All subjects provided written informed consent prior to study entry.

### 4.1. Study Population

Hypercholesterolemic individuals were recruited in the outpatient Unit of Endocrinology and Metabolic Diseases (Parma University Hospital, Italy) between February and June 2016. Eligible subjects were men and women aged ≥18 and ≤75 years, with non HDL-C levels ≥160 mg/dL, having stable weight, dietary habits and physical activity in the three months prior to the screening visit. Key exclusion criteria included diabetes, reduced renal (glomerular filtration rate, GFR < 60 mL/min) or hepatic (transaminase levels >2.5-folds the upper limit of normal values) function, history of cerebro-vascular events, present or past history of alcohol or drug abuse, and neoplastic diseases in the five years prior to study visit. Other exclusion criteria were the use of drugs or food supplements interfering with cholesterol levels, monogenic dyslipidemia, poorly controlled hypothyroidism, and pregnancy.

### 4.2. Study Design

Subjects have been randomly assigned to nutraceutical intervention (containing an association of RRYE titrated in MC 3mg, BC 200mg, CH 10mg and Q10 10 mg) or placebo once daily for 12 weeks, according to a 3:1 randomized block design (block size = 4). Randomization and blinding were provided by DOC Generici Srl (Milano, Italy). The placebo tablets were identical in shape and taste to the nutraceutical compound. Randomization codes were only disclosed after completion of the study and closure of the database. At baseline, family and personal medical history, current therapies, smoking and drinking habits were recorded and physical examination performed. At baseline, 4 and 12 weeks clinical parameters (BMI, waist circumference, systolic and diastolic blood pressure and heart rate) were recorded. At each visit, venous plasma samples were drawn after an overnight fasting for the determination of FPG, HbA1C, plasma insulin, total cholesterol, triglycerides, HDL-cholesterol, liver enzymes (AST, ALT), serum creatinine, CPK, ApoA and ApoB. Non-HDL-C was calculated by subtracting HDL-C from total cholesterol, whereas LDL-C using Friedewald Equation [40]. In addition, circulating pro-inflammatory markers (IL-1, IL-6, IL-10, high-sensitivity CRP (hsCRP), tumor necrosis factor (TNF)-α) levels, hormonal profile [Glucagon-like peptide-1 (GLP-1), Gastric Inhibitory Peptide (GIP) and glucagon] and PCSK9 levels were assessed. EPC number was also determined at baseline and after 12 weeks of treatment.

### 4.3. Coleosoma Technology

The nutraceutical product was assembled with a novel technology (Coleosoma, Labomar Research, International Patent Pending) consisting of a gastro-resistant tablet containing, in the inner core, a CH polymer and an organic acid PS that, once dispersed into the enteric fluids, produces CH cationization. CH is a polyaminosugar not soluble in water except for acidic water solution (pH < 5). Acidic groups protonate amino groups of glucosamine units present in the CH structure, forming a poly-cationic ammonic molecule. Particularly, the acidic molecule selected to project this technology (PS) is a phosphorylated acid capable of cationizing CH and produce irreversible complexes with bile salts. The capability of coleosoma to entrap bile salts and the surrounding mechanism of action, have been investigated and proven in vitro [17].

### 4.4. Hormones and Inflammatory Marker Assessment

Glucagon, GIP, active GLP-1, TNF-α, IL-6, IL-10 and IL-1β circulating concentrations were quantified by a magnetic bead kit (Merck-Millipore, Vimodrone, Italy) according to manufacturer’s instructions and analyzed on a MagPix (Luminex Corporation, Austin, TX, USA). The assay is based on the attachment of the analyte to magnetic beads and processing using LED excitation. Glucagon, GIP and GLP-1 intra- and inter-assay coefficients of variation were: 7% and 11%, 7% and 9%, 8% and 12%, respectively. TNF-α, IL-6, IL-10 and IL-1β intra- and inter-assay coefficients of variation were: 3% and 14%, 4% and 17%, 4% and 15%, 3% and 13%, respectively.

### 4.5. PCSK9 Assessment

PCSK9 plasma levels were assessed by Simple Plex (Bio-Techne, Minneapolis, MN, USA), an automatic enzyme-linked immunosorbent assay (ELISA) system which utilizes microfluidic cartridges with pre-loaded reagents [41]. After thawing, each sample was centrifuged and diluted 1:20 with Sample Diluent prior to assaying. Intra-assay coefficient of variation, calculated in three replicate reactions of one sample, was 2.8%. Inter-assay coefficient of variation, relative to the same sample tested in four different cartridges, resulted of 3.9%.

### 4.6. Quantification of Circulating Endothelial Progenitor Cells

EPC levels were assessed by cytofluorimetric analysis and identified as cells co-expressing CD34, CD133, and Kinase insert Domain Receptor (KDR) surface antigens as previously described [42,43,44,45]. Briefly, 300 µL of blood collected in EDTA were stained with 15 μL fluorescein isothiocyanate (FITC)-conjugated anti-human CD34 monoclonal antibody (mAb) (Becton Dickinson Biosciences, Franklin Lakes, NJ, USA), 20 μL phycoerythrin (PE)-conjugated anti-human KDR mAb (R&D Systems, Minneapolis, MN, USA) and 20 µL allophycocyanin (APC)-conjugated anti-human CD133 mAb (Miltenyi Biotec, San Diego, CA, USA). In the lymphomonocytes, CD34^+^/CD133^+^/KDR^+^ and CD34^+^/KDR^+^ cells were distinguished for the expression of KDR within the CD133^+^/CD34^+^ and CD34^+^ gate, respectively. Circulating EPCs were acquired in a FACSCantoII cytometer, analysed using FACSDiva software (BD Biosciences) and expressed as absolute number of CD133^+^/CD34^+^/KDR^+^ or CD34^+^/KDR^+^ cells per 10^6^ cytofluorimetric events. CD34^+^ and CD133^+^ pluripotent cells were also reported. A single trained operator performed all cytofluorimetric analyses and was blinded to the patients’ allocation.

### 4.7. Study Endpoints

The primary endpoint was the change from baseline values of non-HDL-C after 12 weeks of nutraceutical treatment vs. placebo. The secondary endpoints were the change from baseline values of FPG, BMI, waist circumference, HbA1C, LDL-C and HDL-C, triglycerides, ApoB/ApoA1 ratio, inflammatory markers (IL-1β, IL-6, IL-10, hsCRP, TNF-α), hormone profile (insulin, glucagon, active GLP-1, GIP), PCSK9 levels at 4 and 12 weeks and EPC number at 12 weeks of nutraceutical treatment vs. placebo. In addition, the change of non-HDL-C was evaluated after four weeks of treatment.

### 4.8. Safety Assessments-Adverse Events

Adverse events and SAE along with CPK, AST, ALT and creatinine levels have been recorded at each follow-up visit.

### 4.9. Sample Size Determination

Estimating a mean non-HDL-C reduction of 16 mg/dL in 40% of subjects after 12 weeks [33] with an efficacy of 85–90% [46] an optimal two-stage design was used where the estimated response rate was ≥85% and the null hypothesis response rate was 65.5% or less [47]. Under these assumptions, 13 evaluable patients were to be treated in stage 1 of the study (one-sided, α = 0.05, β = 0.2). At least three responses were required to continue to stage 2 where an additional 21 evaluable patients would be treated (up to a total of 34). Overall, if a total of 26 responses or less were observed, the drug would have been considered non-active. These values have been adjusted to a 3:1 randomized design, including 10 subjects in stage 1 and a total of 30 subjects, with a responder rate of 7 and 23, respectively (software PASS, NCSS Statistical Software, Kaysville, UT, USA).

### 4.10. Statistical Analysis

Continuous variables are expressed as means ± standard deviation (SD) or median (interquartile range IQR) for skewed distributed data. Categorical variables are expressed as frequencies. Skewed data were log-transformed in order to achieve normal distribution as confirmed by the Kolmogorov-Smirnov test. Baseline comparisons of parameters between treatment groups were performed using the *t*-test or Mann-Whitney test where appropriate, and Chi-square for categorical variables. Primary and secondary endpoint analyses were performed using the ITT approach by means of GLM for repeated measures with treatment and time (visits at 4 and 12 weeks) as main factors. Pearson was used to test the correlation between baseline levels of non-HDL-C and its reduction at 12 weeks. A *p*-value ≤ 0.05 was considered significant. Statistical analysis was carried out by using SPSS v. 24 (IBM Statistics).

## 5. Conclusions

In conclusion, the tested nutraceutical formulation was effective in the reduction of non-HDL/LDL-C levels at 4 and 12 weeks thus representing a possible therapeutic strategy in dyslipidemic individuals in primary prevention. High-quality randomized controlled trials are warranted to assure long-term efficacy of nutraceutical on CV morbidity and mortality.

## Figures and Tables

**Table 1:** Baseline characteristics of study subjects.

Variables	Total (*n* = 39)	Placebo (*n* = 9)	Nutraceutical Intervention (*n* = 30)	*p* Value
Age (years)	52 ± 11	52 ± 6	52 ± 12	0.9
Gender *n* (%)	18 M (46) 21 F (54)	3 M (33) 6 F (67)	15 M (50) 15 F (50)	0.38
BMI (kg/m^2^)	27 ± 4	25.8 ± 5.3	27.3 ± 4.5	0.38
Waist circumference (cm)	90 ± 11	88 ± 8	91 ± 12	0.4
Systolic blood pressure (mmHg)	130 ± 14	132 ± 14	130 ± 14	0.65
Diastolic blood pressure (mmHg)	84 ± 8	84 ± 7	84 ± 8	0.84
Heart rate (bpm)	72 ± 9	74 ± 8	71 ± 9	0.44
HbA1c (mmol/mol)	33.9 ± 3.5	33.8 ± 3.9	34.0 ± 3.4	0.89
Fasting plasma glucose (mg/dL)	84 ± 10	82 ± 9	85 ± 11	0.51
Insulin (µU/mL)	8.5 ± 5.1	7.5± 3.5	8.7 ± 5.5	0.78
Total cholesterol (mg/dL)	259 ± 28	267 ± 25	257 ± 28	0.32
HDL cholesterol (mg/dL)	62 ± 13	62 ± 15	62 ± 12	0.96
Non-HDL cholesterol (mg/dL)	197 ± 26	205 ± 21	195 ± 27	0.29
LDL cholesterol (mg/dL)	170 ± 24	179 ± 20	168 ± 25	0.21
Triglycerides (mg/dL)	132 (91–151)	136 (109–151)	131 (91–152)	0.61
Apolipoprotein A (mg/dL)	159.2 ± 19.7	158.9 ± 20.7	159.3 ± 19.7	0.87
Apolipoprotein B (mg/dL)	132.5 ± 15.8	137.1 ± 12.1	131.2 ± 16.7	0.23
Creatinine (mg/dL)	0.70 (0.70–0.90)	0.70 (0.65–0.80)	0.80 (0.68–0.90)	0.28
AST (U/L)	24 (21–28)	24 (22–26)	24 (21–29)	0.67
ALT (U/L)	25 (14–32)	26 (24–33)	23 (14–33)	0.38
CPK (U/L)	108 (77–188)	120 (87–169)	107 (70–204)	0.84
hsCRP (mg/L)	1.06 (0.58–2.08)	1.37 (0.61–2.74)	0.98 (0.55–1.96)	0.51
**Concomitant Medications**	**Total (*n* = 39)**	**Placebo (*n* = 9)**	**Nutraceutical Intervention (*n* = 30)**	***p*** **Value**
Anti-hypertensive *n* (%)	9 (23)	1 (11)	8 (27)	0.33
Thyroxine *n* (%)	9 (23)	2 (22)	7 (23)	0.88

Baseline characteristics of the study subjects expressed as (mean ± standard deviation) or median (interquartile range) for continuous data and *n* (%) for categorical data. (M: males, F: females, BMI: body mass index, HbA1c: Glycated haemoglobin, HDL: high-density lipoprotein; LDL: low-density lipoprotein, AST: aspartate aminotranferase; ALT: alanine aminotranferase; CPK: Creatine phosphokinase, hsCRP: High-sensitivity C-reactive protein).

**Table 2:** Effect of the treatments on metabolic profile.

Variables	Placebo	Nutraceutical Intervention	Treatment Effect (GLM)
HbA1C (mmol/mol)			*p =* 0.89
Baseline	33.8 ± 3.9	34.1 ± 3.0	
4 weeks	32.6 ± 2.0	32.6 ± 3.0	
12 weeks	32.3 ± 3.9	32.4 ± 3.4	
Plasma Glucose (mg/dL)			*p =* 0.89
Baseline	82.0 ± 9.5	84.8 ± 11.2	
4 weeks	89.0 ± 9.3	86.6 ± 9.6	
12 weeks	84.7 ± 8.8	85.5 ± 11.4	
BMI (kg/m^2^)			*p =* 0.20
Baseline	25.8 ± 3.5	27.7 ± 4.5	
4 weeks	25.6 ± 3.47	27.7 ± 4.3	
12 weeks	25.5 ± 3.3	27.7 ± 4.3	
Waist circumference (cm)			*p =* 0.35
Baseline	88 ± 8	91 ± 12	
4 weeks	87 ± 7	91 ± 11	
12 weeks	87 ± 6	90 ± 11	
Non-HDL cholesterol (mg/dL)			*p =* 0.008
Baseline	205 ± 21	195 ± 27	
4 weeks	190 ± 30	166 ± 32	
12 weeks	207 ± 26	165 ± 25	
LDL cholesterol (mg/dL)			*p =* 0.008
Baseline	179 ± 20	168 ± 25	
4 weeks	163 ± 31	138 ± 31	
12 weeks	179 ± 25	136 ± 28	
Apo B (mg/dL)			*p =* 0.02
Baseline	137 ± 12	131 ± 17	
4 weeks	121 ± 21	113 ± 18	
12 weeks	142 ± 15	116 ± 18	
HDL cholesterol (mg/dL)			*p =* 0.98
Baseline	61.8 ± 15.4	62.5 ± 12.3	
4 weeks	61.5 ± 12.6	62.6 ± 13.5	
12 weeks	60.3 ± 12.0	58.9 ± 13.5	
Triglycerides (mg/dL)			*p =* 0.75
Baseline	136(109–151)	131(91–152)	
4 weeks	121(100–160)	104(87–143)	
12 weeks	145(81–179)	119(93–154)	
Apo A/Apo B			*p =* 0.07
Baseline	0.88 ± 0.15	0.84 ± 0.17	
4 weeks	0.82 ± 0.17	0.71 ± 0.16	
12 weeks	0.89 ± 0.15	0.71 ± 0.16	
PCSK9 (ng/mL)			*p =* 0.24
Baseline	250 ± 101	235 ± 87	
4 weeks	326 ± 146	257 ± 107	
12 weeks	293 ± 112	256 ± 108	

The table reports mean ± standard deviation or median (inter quartile range) and *p* values of treatment from generalized linear model (GLM) for repeated measures. (HbA1c: Glycated haemoglobin, BMI: body mass index, HDL: high-density lipoprotein; LDL: low-density lipoprotein, Apo: apolipoprotein, PCSK9: proprotein convertase subtilisin/kexin type 9).

**Table 3:** Safety and tolerability profile of the treatment.

Variables	Placebo	Nutraceutical Intervention	Treatment Effect (GLM)
AST (U/L)			*p =* 0.69
Baseline	24 (22–26)	24 (21–29)	
4 weeks	27 (21–29)	27 (22–30)	
12 weeks	23 (21–29)	26 (22–31)	
ALT (U/L)			*p =* 0.76
Baseline	26 (24–33)	23 (14–33)	
4 weeks	25 (20–29)	25 (17–35)	
12 weeks	22 (17–32)	24 (17–34)	
CPK (U/L)			*p =* 0.92
Baseline	120 (87–169)	107 (70–204)	
4 weeks	113 (86–150)	113 (83–216)	
12 weeks	112 (100–175)	136 (98–207)	
Creatinine (mg/dL)			*p =* 0.24
Baseline	0.70 (0.65–0.80)	0.80 (0.68–0.90)	
4 weeks	0.70 (0.55–0.80)	0.80 (0.70–0.90)	
12 weeks	0.70 (0.70–0.78)	0.80 (0.70–0.93)	
GFR (mL/min/1.73 m^2^)			*p =* 0.34
Baseline	101.62 ± 16.71	98.59 ± 21.23	
4 weeks	108.22 ± 23.64	96.28 ± 17.97	
12 weeks	102.38 ± 24.15	96.48 ± 20.88	

The table reports mean ± standard deviation or median (inter quartile range) and *p* values of treatment from generalized linear model (GLM) for repeated measures. (AST: aspartate aminotranferase; ALT: alanine aminotranferase; CPK: Creatine phosphokinase, GFR: glomerular filtration rate).

## Supplementary Materials

Supplementary materials can be found at www.mdpi.com/1422-0067/18/7/1498/s1.

## Abbreviations

AMPK	AMP-activated protein kinase
APC	Allophycocyanin
APO	Apolipoprotein
BC	Berberine chloride
BMI	Body mass index
CH	Chitosan
CPK	Creatine phosphokinase
CV	Cardiovascular
CVD	Cardiovascular disease
EPC	Endothelial progenitor cell
FITC	Fluorescein isothiocyanate
FPG	Fasting plasma glucose
GFR	Glomerular filtration rate
GIP	Gastric inhibitory peptide
GLM	General linear model
GLP-1	Glucagon-like peptide-1
HbA1C	Glycated haemoglobin
HMG-CoA	3-Hydroxy-3-methyl-glutaryl-CoA
hsCRP	High-sensitivity C-reactive protein
IL	Interleukin
IQR	Interquartile range
ITT	Intention to treat
KDR	Kinase insert domain receptor
LDL-C	Low-density lipoprotein cholesterol
MC	Monacolin
Non-HDL-C	Non-High Density Lipoprotein cholesterol
PCSK9	Proprotein convertase subtilisin/kexin type 9
PE	Phycoerythrin
PS	Phosphoserine
RRYE	Red rice yeast extract
SAE	Serious adverse events
SD	Standard deviation
TNF	Tumor necrosis factor
