# Supplementary material for: Effects of a New Nutraceutical Formulation (Berberine, Red Yeast Rice and Chitosan) on Non-HDL Cholesterol Levels in Individuals with Dyslipidemia: Results from a Randomized, Double Blind, Placebo-Controlled Study
Source: Int J Mol Sci. 2017 Jul 12;18(7):1498. doi: 10.3390/ijms18071498 (PMC5535988; doi:10.3390/ijms18071498)
Supplement: Supplementary file 1 [file ijms-18-01498-s001.pdf]

Effects of a new nutraceutical formulation (berberine, red yeast rice and chitosan) on non-HDL cholesterol levels in individuals with dyslipidemia: results from a randomized, double blind, placebo-controlled study

Valentina Spigoni [1§], Raffaella Aldigeri [1§], Monica Antonini [2], Maria Maddalena Micheli [2], Federica Fantuzzi [1], Andrea Fratter [3], Marzia Pellizzato [4], Eleonora Derlindati [1], Ivana Zavaroni [1, 2], Riccardo C. Bonadonna [1, 2], Alessandra Dei Cas [1, 2*].

Inflammatory markers	**Placebo**	**Nutraceutical intervention**	**Treatment effect (GLM)**
**IL-10 (pg/ml)**			
Baseline	14(10-26)	10(5-16)	p=0.64
4 weeks	17(9-23)	10(6-17	
12 weeks	16(9-18)	10(6-17)	
**IL-1β (pg/ml)**			
Baseline	2.0±1.2	2.1±1.1	p=0.48
4 weeks	2.2±1.0	2.5±1.7	
12 weeks	1.8±0.8	2.4±1.5	
**IL-6 (pg/ml)**			
Baseline	1.9(1.8-2.7)	2.3(1.4-3.6)	p=0.78
4 weeks	2.2(1.7-2.8)	2.3(1.3-3.7)	
12 weeks	2.1(1.4-2.3)	2.7(1.5-3.9)	
**TNF-α (pg/ml)**			
Baseline	4.7±1.4	5.6±1.7	p=0.11
4 weeks	5.1±1.5	6.1±2.6	
12 weeks	4.9±1.0	5.6±1.2	
**hsCRP (mg/l)**			
Baseline	1.4(0.6-2.6)	1.0(0.6-1.9)	p=0.47
4 weeks	0.9(0.7-2.9)	0.9(0.5-1.5)	
12 weeks	0.7(0.6-1.0)	0.9(0.5-1.4)	

Hormones	**Placebo**	**Nutraceutical intervention**	**Treatment effect (GLM)**
**Insulin (μU/ml)**			
Baseline	7(4-11)	8(5-10)	p=0.59
4 weeks	9(7-11)	10(5-13)	
12 weeks	8(4-10)	8(5-13)	
**Active GLP-1 (pg/ml)**			
Baseline	0.6(0.40-3.2)	5.0(0.6-12.9)	p=0.17
4 weeks	0.4(0.4-9.3)	3.0(0.6-13.8)	
12 weeks	2.9(0.6-7.9)	3.0(0.6-12.3)	
**Glucagon (pg/ml)**			
Baseline	37(31-59)	58(40-67)	p=0.20
4 weeks	47(30-60)	58(35-71)	
12 weeks	43(40-46)	58(35-66)	
**GIP (pg/ml)**			
Baseline	78(46-95)	62(43-86)	p=0.70
4 weeks	52(41-79)	74(50-96)	
12 weeks	54(50-95)	81(51-103)	

Table S1. Effect of the treatments on inflammatory and hormone profile. Table S1 reports mean±standard deviation or median (interquartile range) and p values of treatment from general linear model (GLM) for repeated measures. (IL: interleukine; TNF: tumor necrosis factor; hsCRP: high sensitive C reactive protein; GLP-1: glucagon like peptide-1; GIP: Gastric inhibitory peptide)

**Progenitor cells (n/10^6 events)**	**Placebo**	**Nutraceutical intervention**	**Treatment effect (GLM)**
**$KDR^+/CD133^+/CD34^+$**			
Baseline	6(1-10)	4(3-7)	p=0.63
12 weeks	4(2-10)	4(1-7)	
**$KDR^+/CD34^+$**			
Baseline	23(10-46)	11(9-29)	p=0.28
12 weeks	10(6-20)	15(8-17)	
**CD34**			
Baseline	479(320-607)	507(344-622)	p=0.71
12 weeks	431(355-571)	511(334-622)	
**CD133**			
Baseline	343(257-456)	414(263-472)	p=0.66
12 weeks	405(322-542)	415(279-500)	

Table S2. Effect of the treatments on progenitor cell number. Table S2 reports median(interquartile range) and p values of treatment from general linear model (GLM) for repeated measures. (KDR: Kinase insert domain receptor)
